# Job vacancies at the European Centre for Disease Prevention and Control (ECDC)

**DOI:** 10.2807/1560-7917.ES.2020.25.44.2011051

**Published:** 2020-11-05

**Authors:** Eurosurveillance editorial team

**Affiliations:** 1 European Centre for Disease Prevention and Control (ECDC), Stockholm, Sweden

The European Centre for Disease Prevention and Control (ECDC) invites applications for the following positions:

 

**Principal Expert Emergency Preparedness and Response**

The application deadline expires on 24 November 2020. 

 

***Deadline extended*: Scientific editor, *Eurosurveillance***

The application deadline expires on 12 November 2020. 

****

For more information, please visit the ECDC website: 

https://ecdc.europa.eu/en/work-us/vacancies?f%5B0%5D=deadline_date%3A1

